# Hydrogen Sulfide Up-Regulates the Expression of ATP-Binding Cassette Transporter A1 via Promoting Nuclear Translocation of PPARα

**DOI:** 10.3390/ijms17050635

**Published:** 2016-04-29

**Authors:** Dong Li, Qinghui Xiong, Jin Peng, Bin Hu, Wanzhen Li, Yizhun Zhu, Xiaoyan Shen

**Affiliations:** 1 Laboratory of Pharmacology and Toxicology, School of Pharmaceutical Sciences, Sun Yat-sen University, Guangzhou 510006, China; itstiming@outlook.com (D.L.); pengj56@mail2.sysu.edu.cn (J.P.); lwz531lwz@163.com (W.L.); 2 Department of Pharmacology, School of Pharmacy, Fudan University, Shanghai 201210, China; xqh.841011@163.com (Q.X.); weiweide420@163.com (B.H.); YZZhu2013@163.com (Y.Z.); 3 Improvinglife Biological Technology (Shanghai) Co., Ltd., Shanghai 201210, China

**Keywords:** hydrogen sulfide, atherosclerosis, ABCA1, PPARα

## Abstract

ATP binding cassette transporter A1 (ABCA1) plays a key role in atherogenesis. Hydrogen sulfide (H_2_S), a gasotransmitter, has been reported to play an anti-atherosclerotic role. However, the underlying mechanisms are largely unknown. In this study we examined whether and how H_2_S regulates ABCA1 expression. The effect of H_2_S on ABCA1 expression and lipid metabolism were assessed *in vitro* by cultured human hepatoma cell line HepG2, and *in vivo* by ApoE^−/−^ mice with a high-cholesterol diet. NaHS (an exogenous H_2_S donor) treatment significantly increased the expression of ABCA1, ApoA1, and ApoA2 and ameliorated intracellular lipid accumulation in HepG2 cells. Depletion of the endogenous H_2_S generator cystathionine γ-lyase (CSE) by small RNA interference (siRNA) significantly decreased the expression of ABCA1 and resulted in the accumulation of lipids in HepG2 cells. *In vivo* NaHS treatment significantly reduced the serum levels of total cholesterol (TC), triglycerides (TG), and low-density lipoproteins (LDL), diminished atherosclerotic plaque size, and increased hepatic ABCA1 expression in fat-fed ApoE^−/−^ mice. Further study revealed that NaHS upregulated ABCA1 expression by promoting peroxisome proliferator-activated receptor α (PPARα) nuclear translocation. H_2_S up-regulates the expression of ABCA1 by promoting the nuclear translocation of PPARα, providing a fundamental mechanism for the anti-atherogenic activity of H_2_S. H_2_S may be a promising potential drug candidate for the treatment of atherosclerosis.

## 1. Introduction

High-density lipoproteins (HDLs) are known to protect against atherogenesis by transporting excess cholesterol out of macrophages through the ATP-binding cassette transporter A1 (ABCA1)-mediated reverse cholesterol transport [1]. ABCA1 belongs to the ATP-binding cassette family that utilizes the energy of adenosine triphosphate (ATP) hydrolysis to carry cholesterol, phospholipids, and other substrates across membranes [2,3,4]. Mutations in the ABCA1 gene cause Tangier disease and familial hypoalphalipoproteinemia which are characterized by a severe reduction in the amount of HDL, demonstrating ABCA1 as a critical molecule in regulating an initial step of reverse cholesterol transport [5]. Thus, ABCA1 has been considered a potential target in the treatment of atherosclerosis [2,6,7,8].

H_2_S is identified as a gasotransmitter in the cardiovascular system of mammals [9,10]. It is endogenously generated by three distinct enzymatic pathways mediated by cystathionine β-synthase, cystathionine γ-lyase (CSE), and by mercaptopyrucate sulfurtransferase coupled with cysteine aminotransferase [11]. There is plentiful evidence indicating that H_2_S has an anti-atherosclerosis property, however, the underlying mechanisms are largely unknown [12,13,14,15,16,17,18]. Although both of ABCA1 and H_2_S were shown to play a significant role in atherogenesis, there is no information about the correlation between them. In order to study it, HepG2 cells and ApoE^−/−^ mice with a high-cholesterol diet were treated with NaHS (an exogenous H_2_S donor), and the expression and function of ABCA1 were determined. Our results indicate that H_2_S upregulated ABCA1 expression through the promotion of PPARα nuclear translocation.

## 2. Results

### 2.1. NaHS Up-Regulates ABCA1, ApoA1, and ApoA2 Expression and Decreases Lipid Accumulation in HepG2 Cells

**Figure 1:**
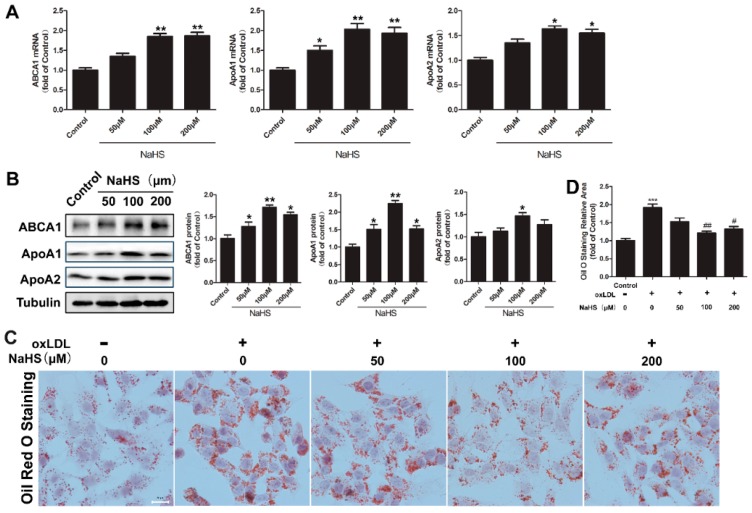
Dosage effect of NaHS on the expression of ABCA1, ApoA1, and ApoA2 in HepG2 cells. (**A**) HepG2 cells were treated with the indicated concentration of NaHS for 6 h, followed by RNA extraction. The relative expression of *ABCA1*, *ApoA1*, and *ApoA2* mRNA was quantified by quantitative reverse transcription polymerase chain reaction (RT-qPCR). Bars indicate means ± SEM of at least three independent experiments (* *p* < 0.05, ** *p* < 0.01 *vs.* control); (**B**) HepG2 cells were treated with the indicated concentration of NaHS for 24 h, followed by total cell lysate preparation. Proteins (20 μg) from total lysates were subjected to SDS-PAGE, followed by Western blot with indicated antibodies. Tubulin was used as a loading control. The blots were then quantified by ImageJ software. (*n* = 3, * *p* < 0.05, ** *p* < 0.01 *vs.* control); (**C**,**D**) HepG2 cells were co-incubated with the indicated concentration of NaHS and oxLDL (50 μg/mL) for 24 h. After incubation, cells were fixed and stained with Oil Red O. Representative images are shown in (**C**). The areas of positive staining were calculated with ImageJ software (*** *p* < 0.001 *vs.* control group; # *p* < 0.05, ## *p* < 0.01 *vs.* oxLDL treated group). Scale bar = 20 μm.

**Figure A1:**
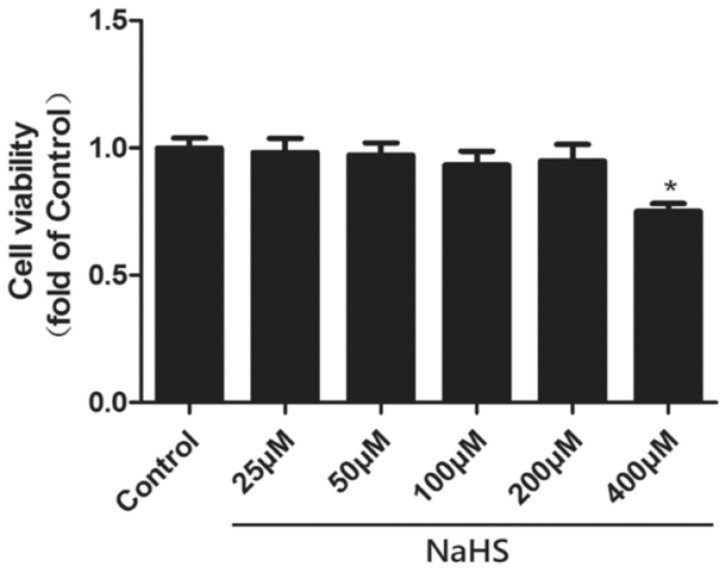
Effect of NaHS treatment on the viability of HepG2 cells. After 12 h of serum starvation, HepG2 cells were treated with the indicated concentrations of NaHS for another 24 h. Cell viability was detected by WST-8 based colorimetric assay. Data were expressed as mean ± SEM. The results are representative of three independent experiments (*n* = 3). * *p* < 0.05 *vs.* control.

In the first set of experiments, the cytotoxicity of NaHS on HepG2 cells was analyzed. HepG2 cells were treated with different doses of NaHS for 24 h. As shown in Figure A1, only 400 μM NaHS treatment exhibited cytotoxicity, the other doses of NaHS had no effect on the viability of HepG2 cells (Figure A1). Therefore, 200 μM of NaHS was used as the highest dose in the following experiments. We then investigated the dose-dependent effect of NaHS on ABCA1 expression. HepG2 cells were treated with 50, 100, or 200 μM NaHS for 6 h (mRNA detection) or 12 h (protein detection), then total RNA and proteins were isolated. As shown in Figure 1A, NaHS up-regulated both the mRNA and protein expression of ABCA1, ApoA1, and ApoA2 in a dose-dependent manner (Figure 1A,B). Especially, the effect of 100 μM NaHS was the most significant.

Based on the fact that NaHS regulates the expression of ABCA1, we assumed that NaHS might play a role in intracellular lipid accumulation. To further explore the effect of NaHS, HepG2 cells were loaded with oxLDL (50 μg/mL) for 24 h in the absence or presence of different doses of NaHS, and the intracellular lipids were stained with Oil Red O. As shown in Figure 1C,D, treatment with 100 or 200 μM NaHS significantly reduced intracellular lipid accumulation. 

### 2.2. Time Course of NaHS Effects on ABCA1, ApoA1, and ApoA2 Gene and Protein Expression

**Figure 2:**
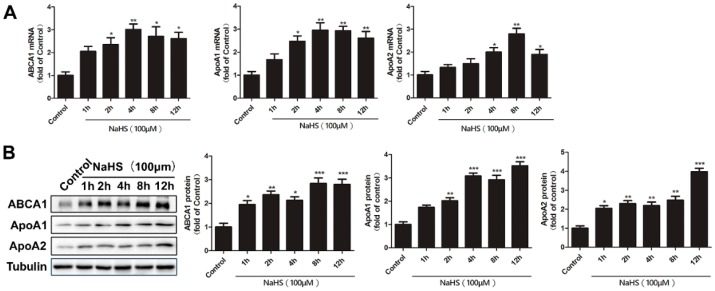
Time course analysis of ABCA1, ApoA1, and ApoA2 expression following NaHS treatment in HepG2 cells. HepG2 cells were treated with 100 μM NaHS for the indicated time periods. (**A**) RNA was extracted and the relative expression of ABCA1, ApoA1, and ApoA2 mRNA was quantified by RT-qPCR. Bars indicate means ± SEM of at least three independent experiments (* *p* < 0.05, ** *p* < 0.01 *vs.* control); (**B**) Total cell lysates were prepared and proteins (20 μg) from total lysates were subjected to SDS-PAGE, followed by Western blot with indicated antibodies. Tubulin was used as a loading control. The blots were then quantified by ImageJ software (*n* = 3, * *p* < 0.05, ** *p* < 0.01, *** *p* < 0.001 *vs.* control).

To understand the time course of changes in *ABCA1*, *ApoA1*, and *ApoA2* gene and protein expression, HepG2 cells were treated with 100 μM NaHS for different amounts of time (1, 2, 4, 8, or 12 h), followed by quantitative reverse transcription polymerase chain reaction (RT-qPCR) and Western blotting. As shown in Figure 2A,B, NaHS treatment increased the *ABCA1*, *ApoA1*, and *ApoA2* mRNA and protein level in a time-dependent manner. Considering the important roles of *ABCA1*, *ApoA1*, and *ApoA2* in lipid metabolism, these results together with the results in Figure 1 indicate an important role of H_2_S in the lipid metabolism of HepG2 cells.

### 2.3. NaHS Alleviated Atherogenesis in High-Fat Dieted ApoE^−/−^ Mice with Increased Liver ABCA1 Expression

**Figure 3:**
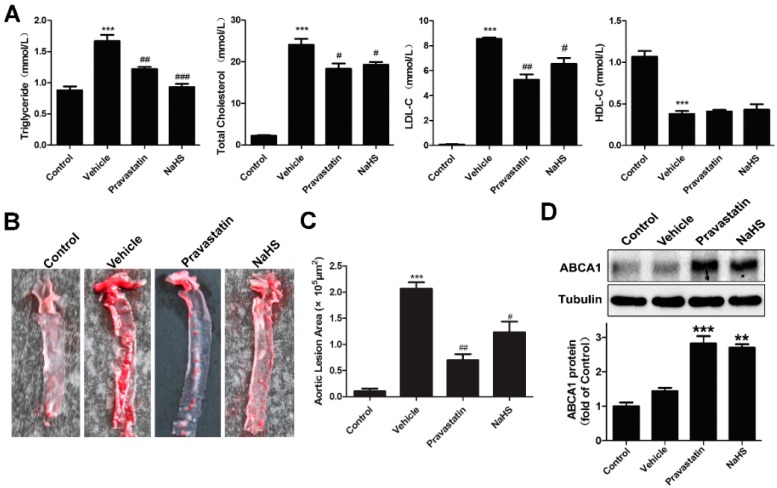
Effects of NaHS on the lipid metabolism of high-fat dieted ApoE^−/−^ mice. Six-week-old ApoE^−/−^ mice fed a high-cholesterol diet were dosed daily with NaHS (50 μM/kg/day, intraperitoneally), pravastatin (30 mg/kg/day, intragastrically), or vehicle (saline solution) for 14 weeks. (**A**) Serum was reserved for detecting total cholesterol (TC), triglycerides (TG), low-density lipoprotein cholesterol (LDL-C) and high-density lipoprotein cholesterol (HDL-C, *n* = 10, *** *p* < 0.001 *vs.* control; ^#^ *p* < 0.05, ^##^ *p* < 0.01, ^###^ *p* < 0.001 *vs.* vehicle); (**B**) Representative images of Oil Red O-stained aortas; (**C**) Quantization of lesion areas in Oil Red O-stained aortas (*n* = 10, *** *p* < 0.001 *vs.* control; ^#^ *p* < 0.05, ^##^ *p* < 0.01 *vs.* vehicle); (**D**) Livers were collected from each group. Equal amount of proteins from liver lysates of each mouse in the same group were pooled and subjected to Western blotting for ABCA1 expression. Tubulin was used as a control (*n* = 10, ** *p* < 0.01, *** *p* < 0.001 *vs.* vehicle).

Previous studies have shown an anti-atherosclerotic property of H_2_S [12,13,14,15,16,17,18]. Apoprotein E (ApoE) is a multifunctional protein that influences many aspects of cardiovascular physiology. ApoE^−/−^ mice have also been extensively utilized as an atherosensitive platform [19]. In the present study, we found that treatment with NaHS (50 μM/kg/day, intraperitoneally) for 14 weeks significantly reduced the serum levels of total cholesterol (TC), triglycerides (TG), and low-density lipoproteins (LDL) induced by high-cholesterol diet (Figure 3A), and alleviated atherogenesis (plaque areas) in ApoE^−/−^ mice fed a high-cholesterol diet (Figure 3B,C, *p* < 0.05). The positive control drug pravastatin (30 mg/kg/day, intragastrically) also had effects similar to NaHS. Since liver ABCA1 plays crucial roles in cholesterol metabolism, we then determined whether NaHS alter the expression of ABCA1. As indicated in Figure 3D, treatment with NaHS increased the protein level of ABCA1 compared with vehicle (*p* < 0.05) in the livers of ApoE^−/−^ mice.

### 2.4. NaHS Upregulated ABCA1 Expression by Promoting PPARα Nuclear Translocation

**Figure 4:**
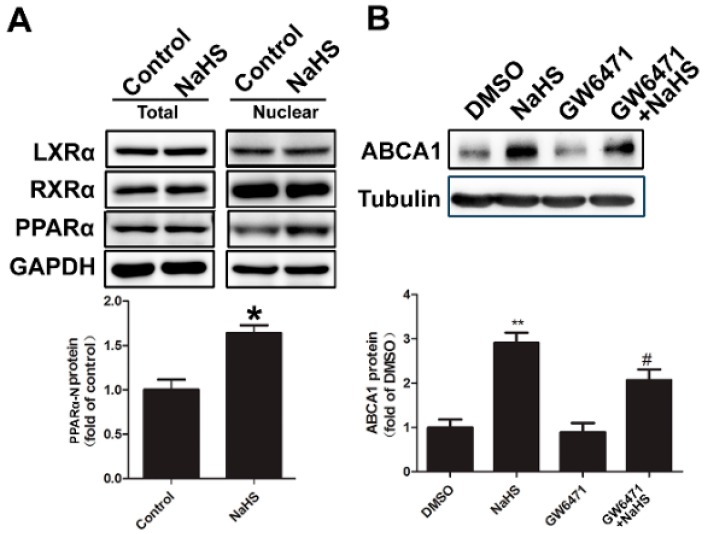
The increase of ABCA1 expression induced by NaHS is mediated by PPARα. (**A**) Total proteins and nuclear proteins of HepG2 cells were prepared after NaHS treatment (100 μM for 2 h). Samples of the proteins were subjected to Western blot with antibodies against LXRα, RXRα, and PPARα. GAPDH were used as the loading controls. The blots of nuclear PPARα were then quantified by ImageJ software (*n* = 3, * *p* < 0.05 *vs.* control); (**B**) HepG2 cells were pretreated with PPARα antagonists GW6471 (25 μM) for 14 h, followed by additional NaHS (100 μM) treatment for 8 h. Cell lysates were collected and probed with anti-ABCA1 and tubulin antibodies (*n* = 3, ** *p* < 0.01 *vs.* DMSO; ^#^ *p* < 0.05 *vs.* NaHS).

To address the nuclear factor involved in the NaHS-induced increase of ABCA1, nuclear proteins were isolated from HepG2 cells after treatment, and the levels of PPARα, LXRα, and RXRα in total lysates and nucleus were compared. As shown in Figure 4A, NaHS treatment increased nuclear level of PPARα, but had no effect on LXRα and RXRα (Figure 4A). To further confirm whether PPARα is involved in NaHS-induced upregulation of ABCA1, pharmacological inhibitors of PPARα (GW6471) were used to pre-treat the HepG2 cells, followed by additional NaHS treatment. In the resting state, GW6471 had no effect on ABCA1 expression (Figure 4B). However, GW6471 can significantly decrease NaHS-induced increase of ABCA1 protein level (Figure 4B), indicating that the increase of ABCA1 expression induced by NaHS might be mediated by PPARα.

### 2.5. CSE Knockdown Decreased the Protein Level of ABCA1 by Inhibiting PPARα Nuclear Translocation

**Figure 5:**
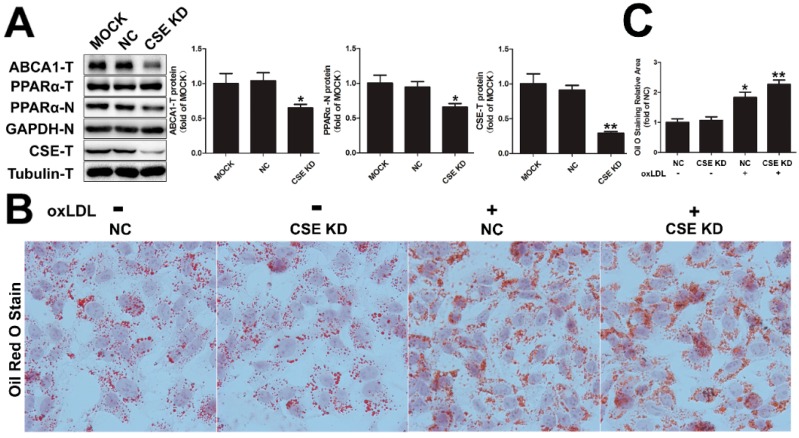
Effect of cystathionine γ-lyase-specific small interfering RNA (CSE-siRNA) on PPARα-meditated ABCA1 expression and lipid accumulation. (**A**) HepG2 cells were transfected without siRNA (MOCK), or with the negative control (NC) or CSE siRNA (CSE KD) for 48 h, total proteins and nuclear proteins were prepared, followed by Western blotting with the indicated antibodies. T: total, N: nuclear. *n* = 3, * *p* < 0.05 and ** *p* < 0.01 *vs.* MOCK; (**B**) HepG2 cells were pretreated with negative control siRNA and CSE siRNA for 24 h, followed by treatment of oxLDL (25 μg/mL) for another 24 h, Scale bar = 20 μm. After incubation, cells were fixed and stained with Oil Red O, and the representative images were shown; (**C**) The areas of positive Oil Red O staining were calculated with ImageJ software (* *p* < 0.01 ** *p* < 0.05 *vs.* NC group).

Hydrogen sulfide is mainly produced by CSE in the liver. To further determine whether the increased ABCA1 expression induced NaHS was achieved by promoting PPARα nuclear translocation, the basal expression of CSE was knocked down in HepG2 cells by cystathionine γ-lyase-specific small interfering RNA (CSE-siRNA). As shown in Figure 5A, CSE knock-down resulted in an obvious reduction of total ABCA1. As expected, the nuclear PPARα was also decreased. This result was also confirmed by Oil Red staining. CSE knock-down by siRNA exacerbated oxLDL accumulation in HepG2 cells (Figure 5B,C). Those results further confirmed an important role of H_2_S in lipid metabolism through PPARα-mediated ABCA1 expression.

## 3. Discussion

ABCA1 is abundant in the liver and plays crucial roles in HDL biogenesis. Studies in hepatic over-expression of ABCA1 have demonstrated that the liver provides the main contribution to the plasma HDL pool [7,8]. More importantly, results from Tangier disease patients and liver-specific ABCA1-knockout mice suggest that hepatic ABCA1 is the most important source of nascent ApoA1 and is absolutely required for the maintenance of the majority of the plasma HDL pool [5,6]. These combined results indicate that ABCA1 is essential in lipid metabolism. In our present study, we used human hepatoma cell line HepG2 and also high-fat dieted ApoE^−/−^ mice as the model systems to identify an important function of hydrogen sulfide in prohibiting lipid accumulation and atherogenesis through upregulation of ABCA1 expression.

Several different mechanisms have been described to be involved in the regulation of ABCA1 expression. Both the mRNA and protein levels of ABCA1 are very unstable, with a half-life of 1–2 h [20], indicating that the transcriptional and translational factors are the major factors for maintaining ABCA1 function. Therefore, the activation and repression mechanisms of ABCA1 function mostly act on the promoter of the ABCA1 gene. Liver-X-receptors (LXR), retinoic acid receptors (RAR), and peroxisome proliferator-activated receptors (PPARs), as well as their coactivators are the most important transcriptional factors for ABCA1 induction [21,22,23,24,25,26]. In the present study, we found that H_2_S upregulates ABCA1 expression by promoting PPARα, but not LXR or RAR nuclear translocation.

Recent studies have suggested that H_2_S plays an anti-atherosclerotic role. Reduction in endogenous H_2_S levels leads to the development and progression of atherosclerosis [12,13,14,15,17,27,28,29,30,31]. Some mechanisms were considered to contribute the anti-atherogenic activity of H_2_S, including the induction of vascular smooth muscle cell apoptosis [32], inhibition of foam cell formation [33], reduction of reactive oxygen species production [34], and adhesion molecule expression [17,18]. However, those findings could not completely explain the anti-atherogenic activity of H_2_S. Gong *et al.* have found that cystathionine γ-lyase (CSE)/hydrogen sulfide could up-regulate ABCA1 and that the PI3K/AKT pathway was involved in the upregulation of ABCA1 expression in THP-1 cells [35]. In the present study, we showed evidence that H_2_S can up-regulate ABCA1 expression through promotion of PPARα nuclear translocation, providing a fundamental mechanism for the anti-atherogenic activity of H_2_S in HepG2 cells and ApoE^−/−^ mice.

PPARs are a group of nuclear receptor proteins that function as transcription factors regulating key metabolic pathways, such as lipid metabolism and inflammation [36]. There are three PPAR isoforms: PPARα, PPARγ, and PPARδ [37,38]. Tissue distribution of PPARs is broad and PPARα is primarily expressed in the liver [37]. In human macrophages, PPARα activation induces the expression of ABCA1, resulting in an increase of cholesterol efflux [39]. PPARα also regulates the synthesis of the major HDL apolipoproteins, ApoA1 and ApoA2, via transcriptional induction [25,40,41]. H_2_S has been reported to hamper the progression of atherosclerosis in fat-fed ApoE^−/−^ mice and down-regulate CX3CR1 and CX3CL1 expression by modulating PPARγ in macrophages and lesion plaques [18]. Our data suggest that the effect of H_2_S on alleviating atherogenesis may be ascribed to increased PPARα nuclear translocation.

It has been reported that nascent HDL particles are a heterogeneous population of different size [42,43]. ABCA1 affects the size of nascent HDL particles by controlling the availability of cell lipids for nascent HDL biogenesis. More importantly, the ABCA1 activity at the cell surface is critical in promoting the initial lipidation of newly-secreted ApoA1, because the lipid-free ApoA1 is not stable and is rapidly cleared from blood through filtration in the kidney [44]. Lipid-poor ApoA1 particles are much more stable than lipid-free ApoA1 particles and can efficiently accept cell cholesterol via ABCA1 [45]. The function of HDL cannot be entirely determined by the plasma concentration. In the present study, we observed that NaHS treatment effectively decreased serum TC, TG, and LDL in fat-fed ApoE^−/−^ mice, but had no effect on HDL levels. Similarly, statins are highly efficacious at lowering low-density lipoprotein cholesterol (LDL-C). However, it has been reported that after 12 weeks of pravastatin treatment, HDL did not change significantly [46]. Whether H_2_S influences lipid-poor ApoA1 formation and the function of HDL are our definite interest and will be clarified in future studies.

## 4. Materials and Methods

### 4.1. Chemicals and Reagents

Sodium hydrosulfide hydrate (NaHS), Dimethyl sulfoxide (DMSO), GW6471, and Oil Red O were purchased from Sigma-Aldrich (St. Louis, MO, USA). Pravastatin sodium tablets were obtained from Daiichi Sankyo Pharmaceutical (Tokyo, Japan). Antibodies against ATP-binding cassette transporter A1 (ABCA1) and Apolipoprotein AI (ApoA1) were obtained from Abcam (Cambridge, UK). Antibodies against Apolipoprotein AII (ApoA2) and PPARα were obtained from Novus (Littleton, CO, USA) and Thermo Fisher (Waltham, MA, USA), respectively. Antibodies against LXRα, RXRα, and CSE were obtained from Santa Cruz (Dallas, TX, USA). Antibodies against Tubulin and GAPDH were obtained from Beyotime (Haimen, China). Anti-mouse and anti-rabbit secondary antibodies were from Promega (Madison, WI, USA).

### 4.2. Cell Culture and NaHS Treatment

Human hepatoma cells line HepG2 cell was obtained from the Type Culture Collection of the Chinese Academy of Sciences (Shanghai, China), maintained in high glucose (25 mM) Dulbecco’s modified Eagle’s medium (DMEM, Thermo Fisher) with 10% (*v*/*v*) fetal bovine serum (Thermo Fisher) at 37 °C under an atmosphere of 5% CO2. The culture plates used for HepG2 cells were coated with Collagen I (2–5 μg/cm^2^, Thermo Fisher) for better adhesion. NaHS was freshly dissolved in DMEM with 10% FBS before adding into the HepG2 cells, and the medium was changed every 12 h with freshly prepared NaHS at the indicated concentration. 

### 4.3. Animals and Biochemical Analysis

All animal experimental procedures were performed in accordance with the “Guide for the Care and Use of Laboratory Animals” (National Institutes of Health) and were approved by the ethics committee of Experimental Research, Shanghai Medical College, Fudan University. ApoE^−/−^ mice (C57BL/6J background, male, SPF) were obtained from the Cavens lab animal Co., Ltd. (Changzhou, China) at 6 weeks of age (License: SCXK (Su) 2011-5003). Mice were divided into four groups (10 mice in each group): the control group (Control; chow-fed, saline solution, intraperitoneally), the model group (Vehicle; high-cholesterol-fed, saline solution, intraperitoneally), pravastatin-treated group (Pravastatin; high-cholesterol-fed, 30 mg/kg/day pravastain, intragastrically), and NaHS treated group (NaHS; high-cholesterol-fed, 50 μM/kg/day NaHS, intraperitoneally) [47,48,49]. The high-cholesterol diet contained 1.25% cholesterol. After 14 weeks, mice were euthanized. Aortas were carefully removed and cleaned of adventitial fat and cut open longitudinally. For detection of atherosclerotic lesions, vessels were fixed by 4% formaldehyde solution for 24 h and stained with Oil Red O solution for 20 min, followed by rinse with isopropanol and distilled water. Images of the stained vessels were acquired by a dissection microscope with a standard digital camera. The lesion area of the stained intimal surface was quantified using ImageJ software, and the data were presented as a percentage of the total surface area covered by Oil Red O staining. Serum was separated and stored at −80 °C until use. Liver samples were quickly excised from the same part, frozen in liquid nitrogen and stored at −80 °C. For measurement of lipid profile, serum total cholesterol (TC), HDL cholesterol (HDL-C), LDL cholesterol (LDL-C), and triglycerides (TG) were determined colorimetrically by commercially available kits supplied by Nanjing Jiancheng Bioengineering Institute (Nanjing, China).

### 4.4. Cell Viability Assay

Cell viability was determined using the Cell Counting Kit-8 (CCK-8) assay (Beyotime). HepG2 cells were planted in 96-well culture plates. After serum starvation for 12 h, the quiescent cells were incubated with NaHS for 24 h. Cell viability was analyzed with the CCK8 kit according to the manufacturer’s instructions. The optical density (OD) of each well was measured at a wavelength of 450 nm.

### 4.5. RNA Isolation and RT-Quantitative-PCR

For RT-qPCR analysis, the cells were lysed in RNAiso Plus reagent (Takara, Kusatsu, Japan) for total RNA extraction, followed by reverse transcription with RevertAid First Strand cDNA Synthesis Kit (Thermo Scientific). Real-Time PCR was performed using SYBR Premix Ex Taq II (Takara) in CFX96TM Real-Time PCR Detection System (Bio-Rad, Hercules, CA, USA). The profile of thermal cycling consisted of initial denaturation at 95 °C for 30 s, and 40 cycles at 95 °C for 5 s and 60 °C for 30 s. All used primers were custom-synthesized by Invitrogen and well-validated. GAPDH was used as an internal control. Sequences of primers ABCA1: 5’-GAGCACAGGCTTTGACCGAT-3’ (forward) and 5’-CTGAGAACCGGCTCTGTTGG-3’ (reverse); ApoA1: 5’-GAGACTGCGAGAAGGAGGTC-3’ (forward) and 5’-CCAGGTCCTTCACTCGATCC-3’ (reverse); ApoA2: 5’-GGAGCTTTGGTTCGGAGACA-3’ (forward) and 5’-TAACCAGTTCCGTTCCAGCC-3’ (reverse); GAPDH: 5’-CTCTGCTCCTCCTGTTCGAC-3’ (forward) and 5’-GCGCCCAATACGACCAAATC-3’ (reverse).

### 4.6. Western Blotting Analysis

Total proteins from the liver of each mouse were isolated and an equal amount of proteins from each mouse in the same group were pooled. After different treatments, the cells were washed twice with cold PBS and lysed in RIPA buffer (Beyotime) on ice. Nuclear extracts were prepared using the Nuclear Extract Kit (Active Motif, Carlsbad, CA, USA), according to the manufacturer’s instructions. An equal amount of proteins from each group were subjected to sodium dodecyl sulfate–polyacrylamide gel electrophoresis (SDS-PAGE), followed by transfer of proteins to nitrocellulose membranes. The membranes were blocked with 5% nonfat milk and incubated with primary antibodies overnight at 4 °C. After incubation with horseradish peroxide-conjugated secondary antibodies, blots were developed with enhanced chemiluminescence (Pierce, MA, USA) and visualized by ChemiDoc™ XRS System from Bio-Rad. The intensity of each lane was quantified by ImageJ software.

### 4.7. Oil Red O Staining

HepG2 cells were co-incubated with the indicated concentration of NaHS and oxLDL (50 μg/mL) for 24 h. The cell monolayer was fixed in 4% (*w*/*v*) paraformaldehyde for 10 min, and then stained by filtered 0.5% Oil Red O. Harris hematoxylin (Beyotime) was used for nuclei counterstaining. Images were acquired by phase-contrast microscopy (Olympus, Tokyo, Japan) from at least five randomly chosen fields for each condition. The areas of positive staining were calculated with ImageJ software.

### 4.8. Small Interfering RNA

The CSE-specific small interfering RNA (siRNA) and negative control siRNA were synthesized by RiboBio (Guangzhou, China). HepG2 cells were transfected with siRNA using Lipofectamine RNAiMAX (Thermo Fisher) according to the manufacturer’s protocol. The cells were subjected to Western blot assay or Oil Red O staining 48 h after transfection.

### 4.9. Statistical Analysis

All values were performed using SPSS software. Intergroup differences were analyzed by one-way ANOVA followed by the Bonferroni *post hoc* analysis for multiple comparison procedure. Unpaired Student’s *t* test was used for comparison between two groups. Differences were considered to be statistically significant when the *p* values were less than 0.05.

## 5. Conclusions

In conclusion, our data supports a therapeutic effect of H_2_S on atherogenesis. In fat-fed ApoE^−/−^ mice, H_2_S decreased serum TC, TG, and LDL level, and up-regulated the expression of hepatic ABCA1. We further demonstrated that the increase of ABCA1 induced by H_2_S was achieved via promoting nuclear translocation of PPARα. Therefore, H_2_S may be a promising potential drug candidate for the treatment of atherogenesis.

## Abbreviations

H_2_S, Hydrogen Sulfide; NaHS, Sodium Hydrosulfide; M, mol/L; ABCA1, ATP binding cassette transporter A1; HepG2, human hepatoma cells line; CSE, cystathionine γ-lyase; TC, total cholesterol; TG, triglycerides; LDL, low-density lipoprotein; HDL, high-density lipoprotein; PPAR, peroxisome proliferator-activated receptor; LXR, liver X receptor; RXR, retinoid X receptor; oxLDL, Oxidized low-density lipoprotein; ApoA1, Apolipoprotein A-I; ApoA2, Apolipoprotein A-II; WST-8, (2-(2-methoxy-4-nitrophenyl)-3-(4-nitrophenyl)-5-(2,4-disulfophenyl)-2*H*-tetrazolium).
